# Supplementary material for: Generation of mesenchyme free intestinal organoids from human induced pluripotent stem cells
Source: Nat Commun. 2020 Jan 10;11:215. doi: 10.1038/s41467-019-13916-6 (PMC6954238; doi:10.1038/s41467-019-13916-6)
Supplement: Supplementary file 7 — Supplementary Information [file 41467_2019_13916_MOESM7_ESM.pdf]

Generation of Mesenchyme Free Intestinal Organoids from Human Induced Pluripotent Stem Cells

Mithal *et al.*

Supplementary Information

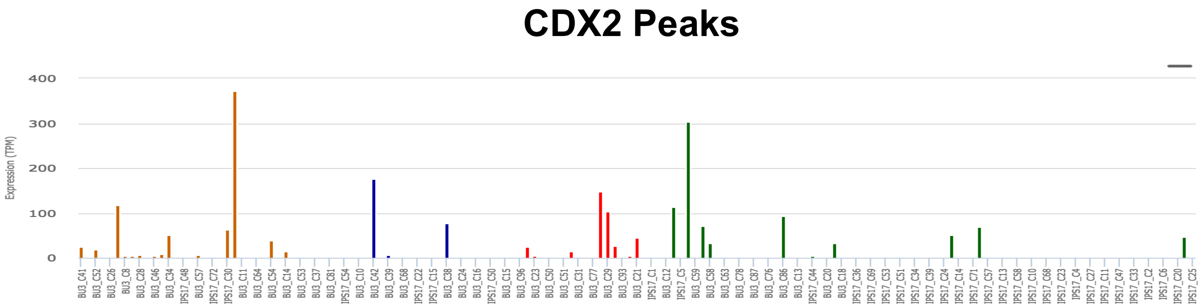

Supplementary Figure 1. Single cell transcriptomics of cells at day 15. Single cell transcriptional profiling of day 15 progenitors reveals a significant number of cells expressing *CDX2*.

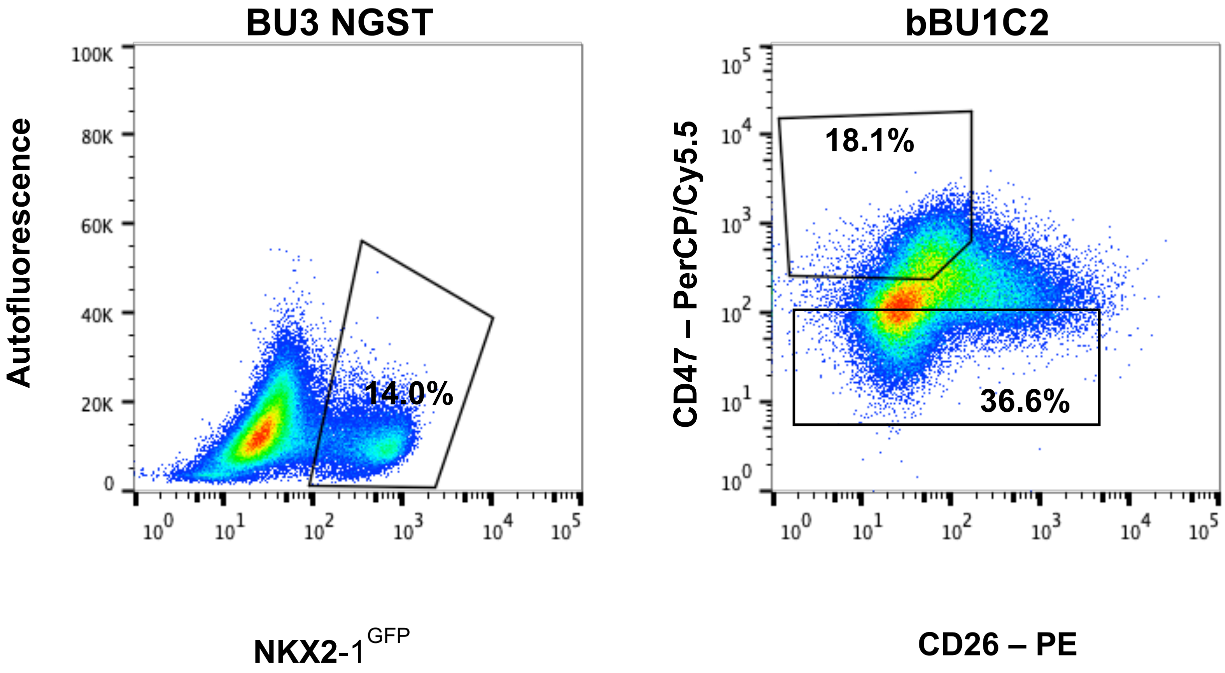

Supplementary Figure 2. Representative FACS plots of day 15 progenitors. Plots of day 15 progenitors from BU3NGST and bBU1C2 differentiations for expression of NKX2-1^{GFP} or CD47/CD26 respectively.

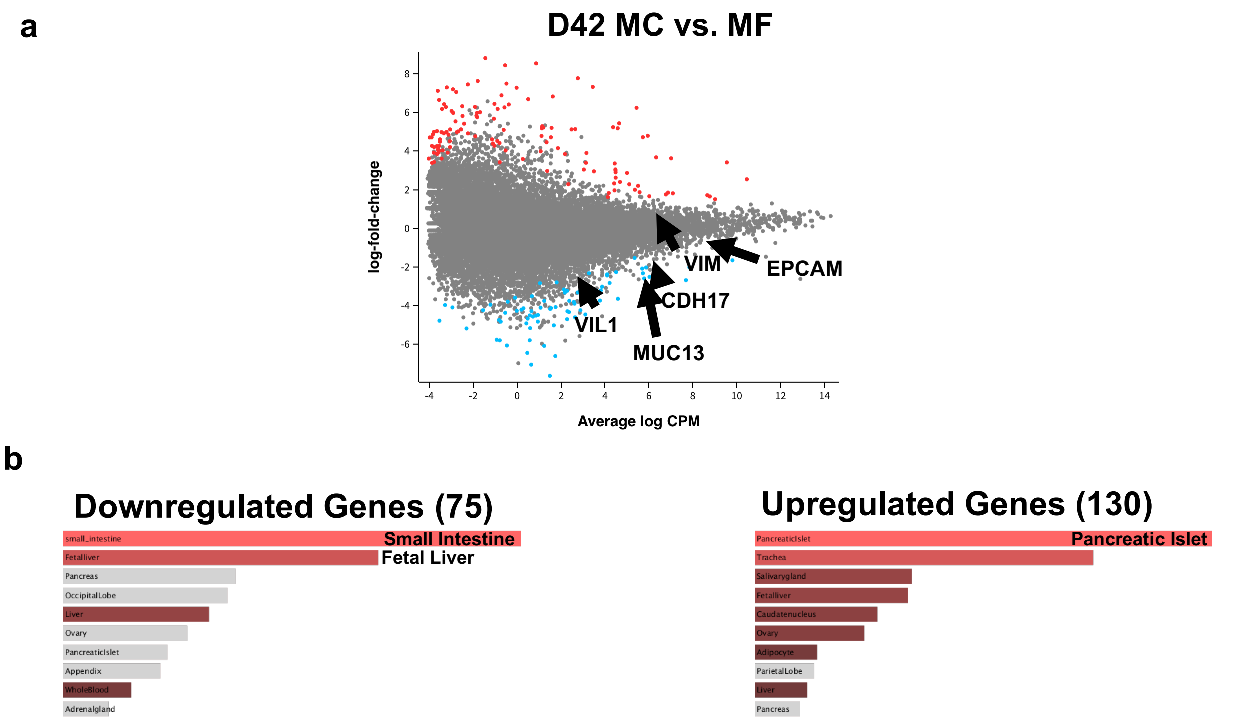

Supplementary Figure 3. RNA-Seq Analysis of D42 HIOs. a Mean Average Plots of significantly differentially expressed genes that were either significantly upregulated (red) or downregulated (blue) in RNA-seq analysis from day 42 MC-derived HIOs as compared to day 42 MF differentiations, both cultured in IM+CHIR. **b** Gene set enrichment analysis using Enrichr showing the top tissue types when referenced to the human gene atlas, Length of red bars indicates combined enrichment score. All colored bars indicate p= <0.05.

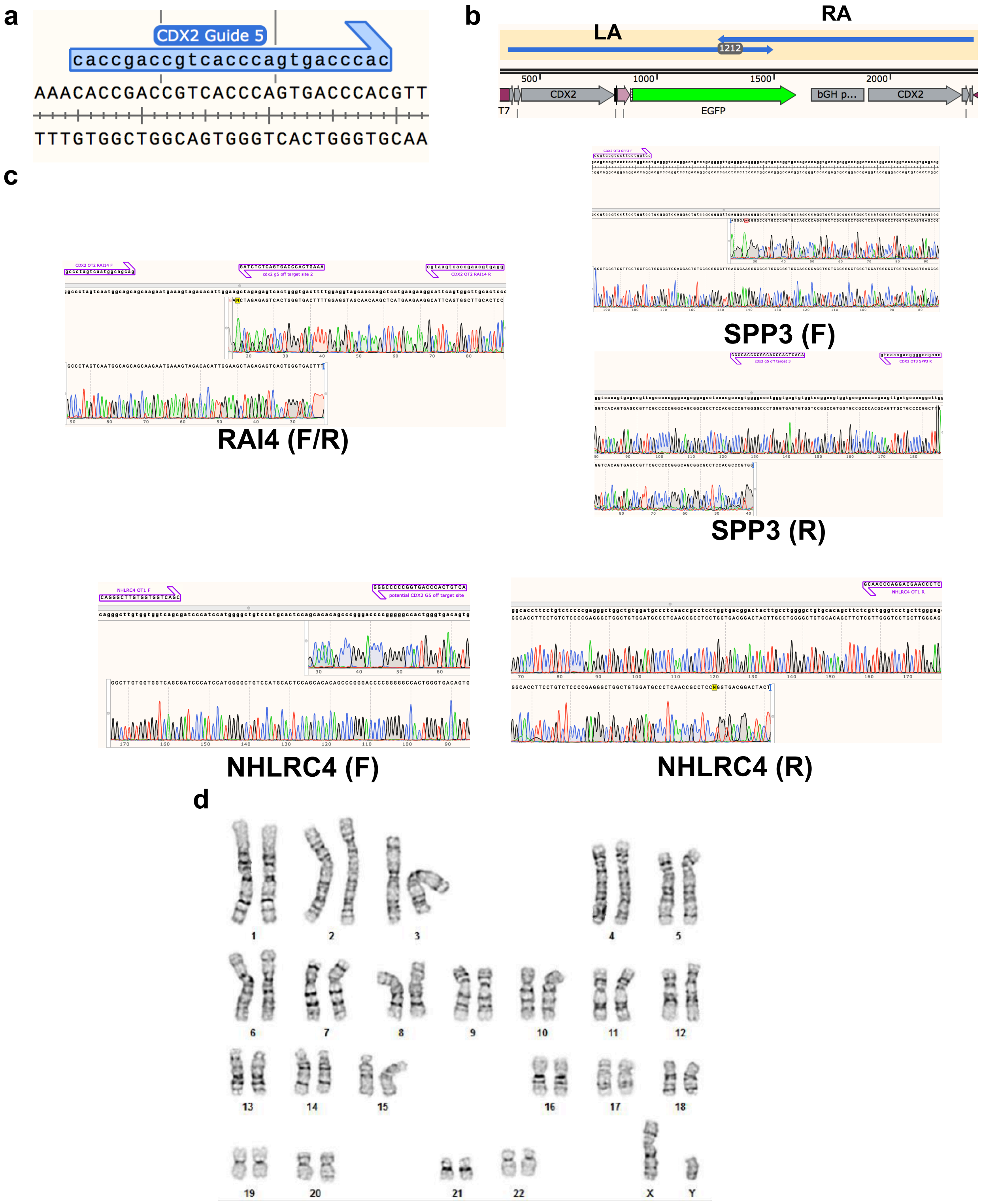

Supplementary Figure 4. Generation and Characterization of bBU1c2-CDX2GFP (BU1CG). a. Sequence and alignment of the Cas9 sgRNA used to gene edit bBU1c2. **b** Sequence alignment of left (LA) and right (RA) arms. **c.** Sequencing for insertion at the three most likely CRISPR off target cut sites based on the G5 sgRNA, NHLRC4, RAI4, and SPP3, with sequencing primers and predicted cut sites shown above. **d.** Normal karyotype of bBU1CG after gene editing.

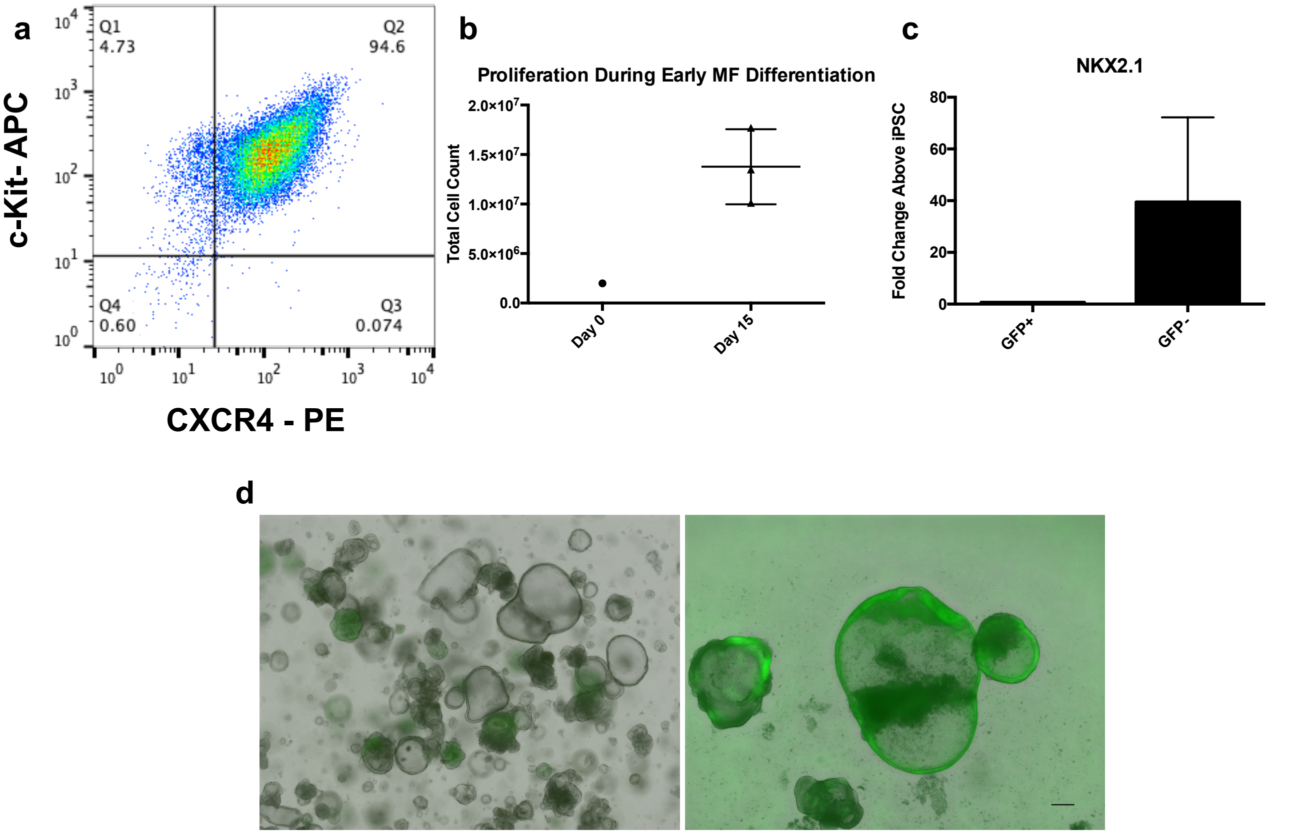

Supplementary Figure 5. NKX2-1 is enriched in the CDX2^{GFP}neg outgrowth. a Representative flow cytometry of Day 3 CXCR4/c-kit$^{+/+}$ definitive endoderm. **b** Proliferation of cells early in the MF differentiation representing average cell output at day 15 per input well of 2 x 10^6 hiPSCs at day 0 (n=3 cell lines) **c** qRT-PCR at day 50 for *NKX2-1* shows that expression of *NKX2-1* tracks with the day 15 CDX2^{GFPneg} sorted outgrowth (n=2, represents $2^{-\Delta\Delta Ct}$, normalized to Day 0 hiPSC and *GAPDH*). **d** Representative micrographs at day 60 show outgrowth that are mostly GFP negative in the GFP- sorted condition (left), as compared to the sustained maintenance of GFP expression in the GFP+ sorted cells (right).

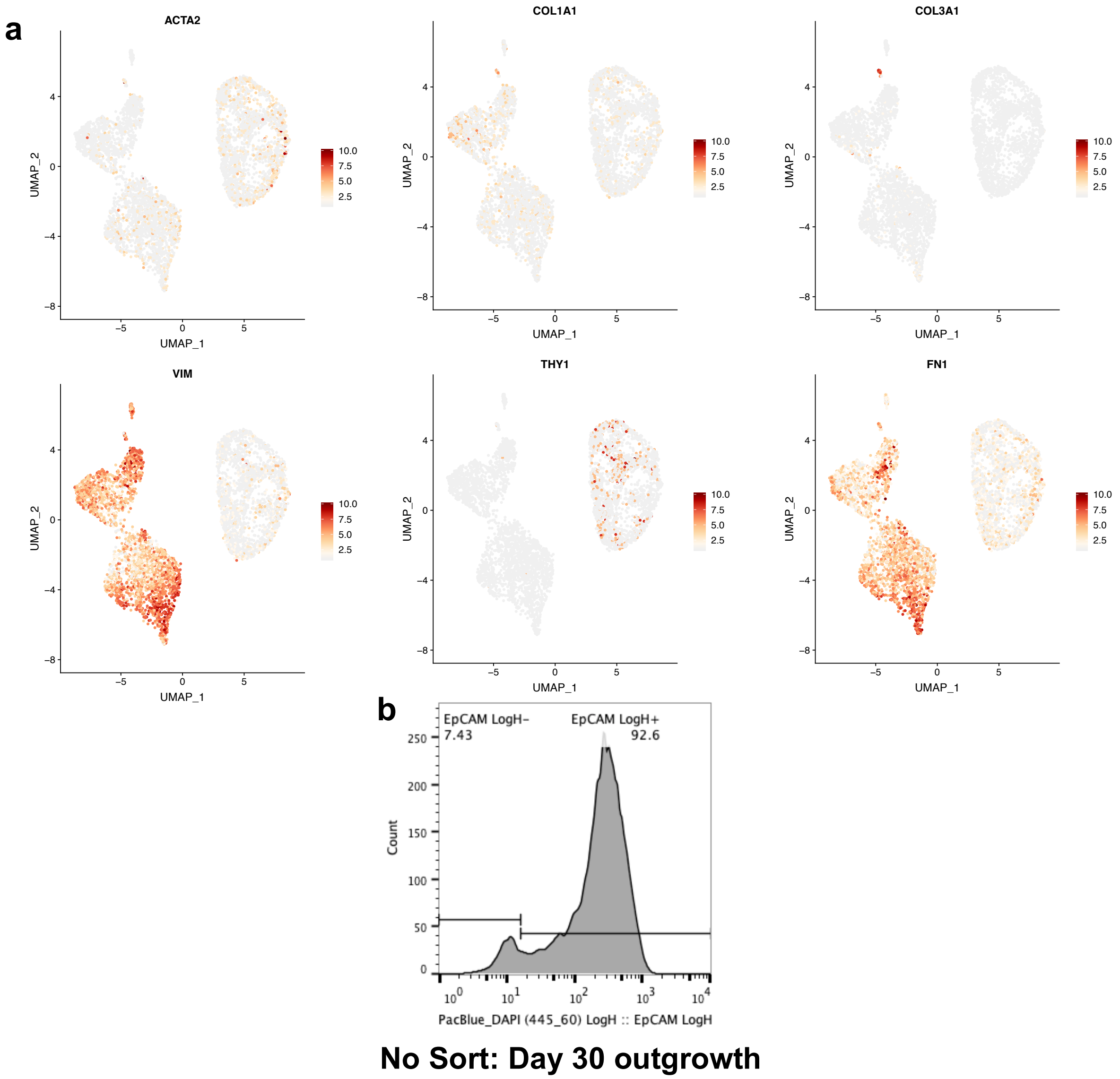

Supplementary Figure 6. Expression Profile of Mesenchymal Genes by scRNAseq. (a) UMAP expression plots of day 6 and day 13 progenitors (as described in Fig. 2) for a variety of mesenchymal-specific genes. (b) Flow cytometry for epithelial-marker EpCAM of D30 outgrowth of the MF protocol in IM+CK, omitting the D15 sorting step.

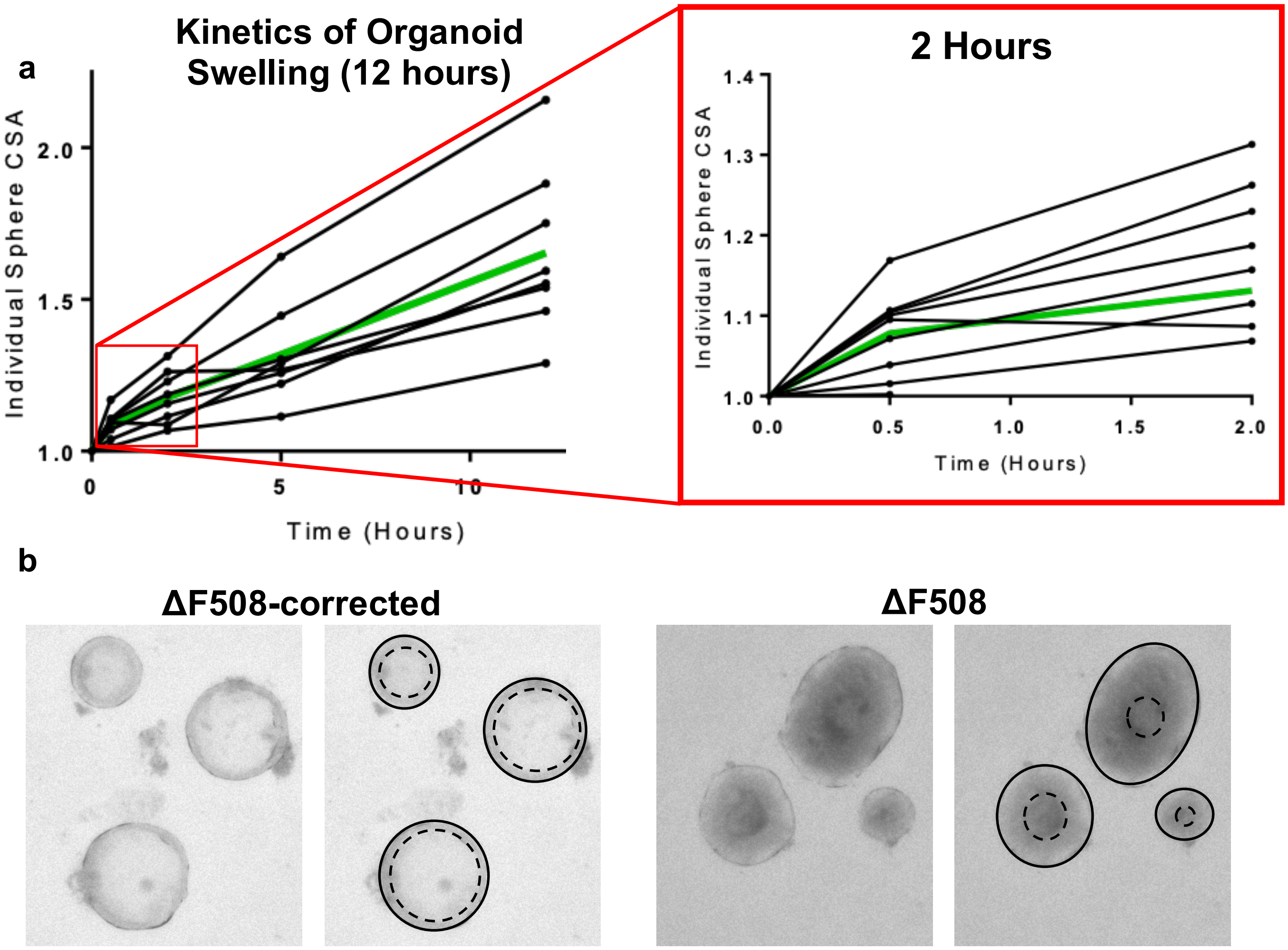

Supplementary Figure 7. Characterization of forskolin-induced HIO swelling. a Kinetics of individual organoid swelling for 12 hours after Forskolin treatment in WT HIOs. Green line indicates mean organoid CSA. **b** Representative images of analysis of SLA in ΔF508 and ΔF508-corrected HIOs (solid line indicates organoid perimeter, dotted line indicates luminal perimeter).

Supplementary Table 1: Growth Factors, Cytokines, and Other Tissue Culture Reagents		
**Name**	**Source**	**Catalog #/ID**
Growth Factor Reduced Matrigel	Corning	356230
Matrigel Basement Membrane Matrix	Corning	354234
SB431542	Tocris	1614
Dorsomorphin	Stemgent	04-0024
CHIR99021	Tocris	4423
Recombinant human FGF10	R&D Systems	345-FG-025
Recombinant human KGF	R&D Systems	251-KG-010
Recombinant human BMP4	R&D Systems	314-BP
Retinoic acid	Sigma	R2625
Y-27632 dihydrochloride	Tocris	1254
Dexamethasone	Sigma	D4902
8-bromoadenosine 3′,5′-cyclic monophosphate sodium salt (cAMP)	Sigma	B7880
3-Isobutyl-1-methylxanthine (IBMX)	Sigma	I5879
Hoechst	Thermo Fisher	H3570
Recmbinant Human FGF4	R&D Systems	235-F4-025
0.05% trypsin-EDTA	Invitrogen	25300-120
Defined Fetal Bovine Serum	Thermo Fisher	NC0652331
Recombinant human Noggin	R&D Systems	6057NG025
Recombinant human EGF	R&D Systems	236EG200
Recombinant Human R-Spondin 1 Protein	R&D Systems	4645-RS-025
N2 Supplement	Invitrogen	17502-048
B27 Supplement	Invitrogen	12587-010
GlutaMAX™	Thermo Fisher	35050061
Gentle Cell Dissociation Reagent	StemCell Technologies	07174
Ascorbic Acid	Sigma	A4403
Cell Recovery Solution	Corning	354253
Paraformaldehyde	Electron Microscopy Sciences	19208
ReLeSR	StemCell Technologies	05873
StemDiff Definitive Endoderm Kit	StemCell Technologies	05110
Accumax™	Sigma	A7089

Supplementary Table 2: Media Recipes/Composition			
**Media**	**Base**	**Cytokines/Growth Factors**	**Concentration**
**IM+CHIR**	DMEM F/12	Noggin	500ng/mL
	Primocin (100ng/mL)	R-Spondin	100ng/mL
	B27	EGF	100ng/mL
	HEPES	CHIR99021	3μM
	Glutamax (100x)		
	N2		
**IM+CK**	DMEM F/12	Noggin	500ng/mL
	Primocin (100ng/mL)	R-Spondin	100ng/mL
	B27	EGF	100ng/mL
	HEPES	CHIR99021	3 μM
	Glutamax (100x)	KGF/FGF7	10ng/mL
	N2		
**Hindgut Media**	RPMI-1640	CHIR99021	3μM
	Fetal Bovine Serum (2%)	FGF4	500ng/mL
**Complete Serum Free Differentiation Medium (csFDM)**	IMDM (75%)		
	Ham's F/12 (25%)		
	B27 (with RA)		
	N2		
	0.05% BSA		
	Primocin (100ng/mL)		
	Glutamax (100x)		
	Ascorbic Acid (50ug/mL)		
	MTG (0.45mM)		
**CK+DCI**	cSFDM	CHIR99021	3μM
		KGF/FGF7	10ng/mL
		Dexamethasone	50nM
		cAMP	0.1mM
		3-isobutyl-1-methyxanthine [IBMX]	0.1mM

Supplementary Table 3: Sequence Based Reagents	
**Description**	**Cat No**
**Taqman Probes**	
CDH17	Hs00900408_m1
GAPDH	Hs99999905_m1
CDX2	Hs01078080_m1
PDX1	Hs00426216_m1
LYZ	Hs00426232_m1
ALB	Hs00609411_m1
PGC	Hs00160052_m1
CFTR	Hs00357011_m1
VIL1	Hs00200229_m1
GATA4	Hs00171403_m1
**Miscellaneous**	
Human Colon RNA	ThermoFisher Scientific: AM7986
**SYBR Green Primers (IDT)**	
SATB2 F	CCACCTTCCCAGCTTGATT
SATB2 R	TTAGCCAGCTGGTGGAGACT
MUC2 F	CCTTGAACGCAAAGTGGAATC
MUC2 R	GACATGCTGTTCCTGAATCTGAG
BACTIN F	CATGTACGTTGCTATCCAGGC
BACTIN R	CTCCTTAATGTCACGCACGAT
VIMF	CCTTGAACGCAAAGTGGAATC
VIMR	GACATGCTGTTCCTGAATCTGAG
**CDX2 eGFP Knock-In**	
spCAS9 G5F	CACCGACCGTCACCCAGTGACCCAC
spCAS9 G5R	AAACGTGGGTCACTGGGTGACGGTC
CDX2 INSERT TEST F	AGCCTCCTTCTACCTTTAGTCC
CDX2 INSERT TEST R	AGCACCTGGCCATTCAGATG
CDX2 OT1 NHLRC4 F	CAGGGCTTGTGGTGGTCAGC
CDX2 OT1 NHLRC4 R	CTCCCAAGCAGGACCCAACG
CDX2 OT2 RAI14 F	GCCCTAGTCAATGGCAGCAG
CDX2 OT2 RAI14 R	GGAGTGCAAGCCACTGAATGC
CDX2 OT3 SPP3 F	CCGTCCGTCCTTCCTGGTCC
CDX2 OT3 SPP3 R	CAAGCCGGGGCAGCAACTG

Supplementary Table 4: Antibodies/Dyes, and Reagents for Immunostaining		
**Target**	**Vendor**	**Catalog No**
Anti-human CD47 PerCP/Cy5.5 Conjugate	BioLegend	323110
Anti-human CD26 PE Conjugate	BioLegend	302705
Calcein Blue	Life Technologies	C1429
Anti-human CXCR4 - PE	ThermoFisher	MHCXCR404
Anti-human c-Kit - APC	BioLegend	323205
Donkey serum	Jackson Immunoresearch Labs	017-000-121
Donkey anti-goat DyLight 488	ThermoFisher Scientific	SA5-10086
Cy™3 AffiniPure Donkey Anti-Rabbit	Jackson Immunoresearch Labs	711-165-152
Donkey anti-Mouse IgG DyLight 488	ThermoFisher Scientific	SA5-10166
13 mm cover slips	ThermoFisher Scientific	174950
Fluoromont -G	Southern Biotech	0100-01
Rabbit anti-CDX2	ThermoFisher Scientific	MA5-14494
Anti-chromogranin A	abcam	ab15160
Anti-Vimentin antibody	abcam	ab8978
HistoGel™ Specimen Processing Gel	Richard Allen Scientific	11330057
Mouse anti-Villin	Millipore	MAB1671
Hoechst 33342	ThermoFisher Scientific	62249
Anti- MUC2	SantaCruz	sc-515032
Anti- Lyz	ThermoFisher Scientific	PA1-29680
Chicken Anti- GFP IgY	ThermoFisher Scientific	A10262
Donkey anti-chicken AF488	Jackson Immunoresearch Labs	703-545-155
