# Supplementary material for: Generation of mesenchyme free intestinal organoids from human induced pluripotent stem cells
Source: Nat Commun. 2020 Jan 10;11:215. doi: 10.1038/s41467-019-13916-6 (PMC6954238; doi:10.1038/s41467-019-13916-6)
Supplement: Supplementary file 9 — Reporting Summary [file 41467_2019_13916_MOESM9_ESM.pdf]

nature research

Corresponding author(s): Gustavo Mostoslavsky

Last updated by author(s): 11-13-2019

Reporting Summary

Nature Research wishes to improve the reproducibility of the work that we publish. This form provides structure for consistency and transparency in reporting. For further information on Nature Research policies, see Authors & Referees and the Editorial Policy Checklist.

Statistics

For all statistical analyses, confirm that the following items are present in the figure legend, table legend, main text, or Methods section.

n/a	Confirmed	
☐	☒	The exact sample size (*n*) for each experimental group/condition, given as a discrete number and unit of measurement
☐	☒	A statement on whether measurements were taken from distinct samples or whether the same sample was measured repeatedly
☐	☒	The statistical test(s) used AND whether they are one- or two-sided<br>*Only common tests should be described solely by name; describe more complex techniques in the Methods section.*
☐	☒	A description of all covariates tested
☒	☐	A description of any assumptions or corrections, such as tests of normality and adjustment for multiple comparisons
☐	☒	A full description of the statistical parameters including central tendency (e.g. means) or other basic estimates (e.g. regression coefficient) AND variation (e.g. standard deviation) or associated estimates of uncertainty (e.g. confidence intervals)
☒	☐	For null hypothesis testing, the test statistic (e.g. *F*, *t*, *r*) with confidence intervals, effect sizes, degrees of freedom and *P* value noted<br>*Give P values as exact values whenever suitable.*
☒	☐	For Bayesian analysis, information on the choice of priors and Markov chain Monte Carlo settings
☐	☒	For hierarchical and complex designs, identification of the appropriate level for tests and full reporting of outcomes
☒	☐	Estimates of effect sizes (e.g. Cohen's *d*, Pearson's *r*), indicating how they were calculated

Our web collection on statistics for biologists contains articles on many of the points above.

Software and code

Policy information about availability of computer code

Data collection	CellCapTure, Moflo Astrios software from Beckman Coulter, and BD CellQuest Pro (TM) were used to collect flow cytometry data. FlowJo was used to analyze Flow Cytometry data. Software from 10x Genomics and Illumina were used to capture library preparation and sequencing data. The QuantStudio 6 real time PCR software was used to capture qRT-PCR data.
Data analysis	For the Bulk RNA sequencing experiments in Fig. 1, we used edgeR package (ref 83) to import, filter and normalize the count matrix, followed by the glimma package (ref 84) and voom (ref 85) for linear modeling and differential expression testing using empirical Bayes moderation to estimate gene-wise variability before significance testing based on the moderated t-statistic. Gene set enrichment analysis was performed using Enrichr (refs 28 and 29).<br>For the scRNAseq experiment, the Cell Ranger software pipeline produced the FASTQ and Counts matrix files. Seurat ver. 3.0 was used to further process data

For manuscripts utilizing custom algorithms or software that are central to the research but not yet described in published literature, software must be made available to editors/reviewers. We strongly encourage code deposition in a community repository (e.g. GitHub). See the Nature Research guidelines for submitting code & software for further information.

Data

Policy information about availability of data

All manuscripts must include a data availability statement. This statement should provide the following information, where applicable:

- Accession codes, unique identifiers, or web links for publicly available datasets
- A list of figures that have associated raw data
- A description of any restrictions on data availability

The dataset supporting the conclusions of the sc-RNAseq experiment (Fig. 2) is available in the GEO repository, accession GSE140405 in hhttps://www.ncbi.nlm.nih.gov/geo/query/acc.cgi?acc=GSE140405. The dataset supporting the conclusions of the bulk RNA-sequencing experiment (Fig. 1) is available in the GEO repository, accession GSE128922 in https://www.ncbi.nlm.nih.gov/geo/query/acc.cgi?acc=GSE128922. The remainder of the data that support the findings of this study are available from the corresponding author upon reasonable request.

Further details of iPSC derivation, characterization, and culture are available for free download at http://www.bu.edu/dbin/stemcells/protocols.php.

Field-specific reporting

Please select the one below that is the best fit for your research. If you are not sure, read the appropriate sections before making your selection.

☒ Life sciences ☐ Behavioural & social sciences ☐ Ecological, evolutionary & environmental sciences

For a reference copy of the document with all sections, see nature.com/documents/nr-reporting-summary-flat.pdf

Life sciences study design

All studies must disclose on these points even when the disclosure is negative.

Sample size	Sample size of n=3 was chosen to ensure both feasibility and reproducibility. Each differentiation takes 40+ days to complete, and so an n=3 in each cell line was determined to be an adequate sample in order to allow for reasonable completion of experiments while also ensuring that the results are reproducible across multiple genetic backgrounds.
Data exclusions	N/A
Replication	Independent experiments (n's) represent independent directed differentiations of the same cell lines. We have also been able to replicate our findings across at least four cell lines, as described in the manuscript.
Randomization	N/A
Blinding	N/A

Reporting for specific materials, systems and methods

We require information from authors about some types of materials, experimental systems and methods used in many studies. Here, indicate whether each material, system or method listed is relevant to your study. If you are not sure if a list item applies to your research, read the appropriate section before selecting a response.

Materials & experimental systems

n/a	Involved in the study	
☐	☒	Antibodies
☐	☒	Eukaryotic cell lines
☒	☐	Palaeontology
☒	☐	Animals and other organisms
☒	☐	Human research participants
☒	☐	Clinical data

Methods

n/a	Involved in the study	
☒	☐	ChIP-seq
☐	☒	Flow cytometry
☒	☐	MRI-based neuroimaging

Antibodies

Antibodies used	Listed in Supplementary Material
Validation	Provided by manufacturers

Eukaryotic cell lines

Policy information about cell lines

Cell line source(s)	CReM iPSC Core Facility (in-house)
Authentication	Cell lines were karyotyped after reprogramming, and determined to have normal karyotypes by the CReM iPSC core.
Mycoplasma contamination	All cell lines were tested for mycoplasma contamination. The CReM performs mycoplasma surveillance screening every six months. No positive tests were recorded during the completion of the experiments described within the manuscript.
Commonly misidentified lines<br>(See ICLAC register)	N/A

Flow Cytometry

Plots

Confirm that:

☒ The axis labels state the marker and fluorochrome used (e.g. CD4-FITC).

☒ The axis scales are clearly visible. Include numbers along axes only for bottom left plot of group (a 'group' is an analysis of identical markers).

☒ All plots are contour plots with outliers or pseudocolor plots.

☒ A numerical value for number of cells or percentage (with statistics) is provided.

Methodology

Sample preparation	Cells were removed from tissue culture plates using either gentle cell dissociation reagent (GCDR) or 0.05% trypsin. Cells were spun down at 300 x g for 5 minutes, stained with antibodies in Flow Buffer (PBS + 0.5%FBS) at room temperature protected from light for 20-30 minutes, washed again at 300 x g, and resuspended in final staining buffer (supplemented with Y27632 for sorting).
Instrument	Stratedigm<br>BD FACSCalibur<br>MoFlo Astrios
Software	FlowJo (FlowJo LLC)
Cell population abundance	Sorting and purity determinations are described in the manuscript extensively.
Gating strategy	FSC/SSC gating were drawn to include single cells. All other gating strategies were determined by an isotype, undifferentiated (for fluorescent reporters), or an unstained negative control.

☒ Tick this box to confirm that a figure exemplifying the gating strategy is provided in the Supplementary Information.
