# Supplementary material for: Generation of mesenchyme free intestinal organoids from human induced pluripotent stem cells
Source: Nat Commun. 2020 Jan 10;11:215. doi: 10.1038/s41467-019-13916-6 (PMC6954238; doi:10.1038/s41467-019-13916-6)
Supplement: Supplementary file 10 — Description of Additional Supplementary Files [file 41467_2019_13916_MOESM10_ESM.pdf]

Title: Supplementary Movie 1:
Description: 19-hour time lapse of BU1CG (Control) HIOs treated with forskolin

Title: Supplementary Movie 2:
Description: 19-hour time lapse of BU1CG (Control) HIOs treated with DMSO

Title: Supplementary Movie 3:
Description: 19-hour time lapse of RC204 (ΔF508 uncorrected) HIOS treated with forskolin

Title: Supplementary Movie 4:
Description: 19-hour time lapse of RC204 (ΔF508 uncorrected) HIOs treated with DMSO

Title: Supplementary Movie 5:
Description: 19-hour time lapse of RC204-corr (ΔF508 corrected) HIOs treated with forskolin

Title: Supplementary Movie 6:
Description: 19-hour time lapse of RC204-corr (ΔF508 corrected) HIOs treated with DMSO Scale Bar = 250µm, Time scale indicates elapsed time from t = 0hr.
